# Association of human platelet alloantigens encoding gene polymorphisms with the risk of Coronary artery disease in Iranian patients

**DOI:** 10.1186/s12872-021-01892-z

**Published:** 2021-02-02

**Authors:** Farideh Malakootikhah, Hossein Naghavi, Negar Firouzabadi, Mohsen Maadani, Massoumeh Shafiei, Nader Tajik

**Affiliations:** 1 Immunology Research Center (IRC), Institute of Immunology and Infectious Disease, Iran University of Medical Sciences, Shahid Hemmat Highway 14496, Tehran, Iran; 2 Department of Immunology, School of Medicine, Tehran University of Medical Sciences, Tehran, Iran; 3 Department of Pharmacology and Toxicology, School of Pharmacy, Shiraz University of Medical Sciences, Shiraz, Iran; 4 Cardiovascular Intervention Research Center, Shaheed Rajaie Cardiovascular Medical and Research Center, Iran University of Medical Sciences, Tehran, Iran; 5 Department of Pharmacology, School of Medicine, Iran University of Medical Sciences, Tehran, Iran

**Keywords:** Coronary artery disease, Human platelet alloantigens, Platelet aggregation, Haplotype, PCR-SSP

## Abstract

**Background:**

Coronary artery disease (CAD) is characterized by narrowing/ blockade of coronary arteries that is mainly caused by atherosclerotic plaques. Considering the involvement of platelet abnormalities, such as defective aggregation and adhesion, in the cardiovascular-related disorders, genetic variations in human platelet alloantigens (HPA) have been implicated in the CAD susceptibility. Herein, we intended to determine the association of HPA-1 to -6, -9, and -15 biallelic polymorphisms with CAD in an Iranian population.

**Methods:**

In this retrospective case–control study, 200 CAD subjects and 100 matched healthy individuals were enrolled. DNA samples were isolated from peripheral blood samples and genotyping of HPA polymorphisms was accomplished using polymerase chain reaction-sequence-specific primers.

**Results:**

The alleles and genotypes of studied HPA polymorphisms were equally distributed among cases and controls and therefore no statistically significant differences were detected. Univariate analysis identified no association of combined haplotypes with CAD risk. However, multivariate analysis showed a positive association of the‌ HPA1b/2a/3b haplotype with CAD after adjustment for some covariates (including BMI, TG, LDL, FBS and blood pressure) that conferred a CAD susceptibility haplotype (*P* = 0.015; OR = 2.792; 95% CI 1.45–8.59).

**Conclusions:**

Although alleles, genotypes, and haplotypes of HPA polymorphisms were not associated with CAD risk, HPA1b/2a/3b haplotype was found to be a dependent disease risk haplotype in Iranian population after correcting for confounding factors.

## Background

Coronary artery disease (CAD) is a leading cause of mortality and morbidity in the globe. According to a report in 2014, about 17.5 million individuals died due to CAD [1], in which approximately 80% of death cases occurred in the countries with low/middle-income economy [2]. CAD in considered as a common disease of the vascular system and is defined by prolonged atherosclerotic lesions that eventuate in narrowing and ultimately obstructing the coronary arteries, resulting in tissue injury. The most critical manifestations of the CAD are myocardial infarction (MI) and angina, with the ultimate development of thrombosis of coronary arteries and rupture of the plaques [3]. Currently, many risk factors have been reported to be involved in susceptibility to CAD. More than 250 genes play critical roles in CAD predisposition that are involved in increasing or decreasing risks of CAD [4–6].

Evidence show that impaired aggregation and adhesion of platelets might be underlying contributing factor in the progression of coronary syndrome. Additionally, genetic polymorphisms in human platelet antigens (HPAs) have been recognized to be associated with susceptibility to CAD [7]. Receptors of the membrane glycoproteins (GP) on the platelets are involved in the activation, aggregation, and adhesion of platelets, the serialized occurrences that culminate in thrombus formation and development of acute coronary syndrome (ACS) [8–10]. Most of the known HPA (20 of 33) are located on the GPIIb/IIIa complex, while the remaining 13 HPAs are expressed on other GP complexes of the platelet, including GPIb/IX/V, GPIa/IIa and CD109 [11]. Several studies have indicated an association between multiple genetic polymorphisms harbored by the genes coding the platelet GPs with dysregulated functions of the platelets, such as promoted platelet adhesion and aggregation, and therefore increase the risk of CAD [12].

Studies have reported the role of HPA-1, HPA-2, and HPA-3 in development of disorders associated with blood clotting and platelet aggregation, hence conferring a risk for the onset of cardiovascular diseases [11]. In addition, most of the GPs on the platelets (such as GPIIb/IIIa) have been shown to harbor these SNPs [13].

Previous studies are inconsistent with the association of HPA-1b/2a/3b alleles, genotypes, haplotypes, and traditional risk factors with CAD. The observed conflict in the results of different studies may stem from diagnostic criteria in determination of the CAD patients, genetic detection techniques, and adjustment of genetic polymorphisms to other risk factors of CAD. Moreover, differences in genetic stratifications in different ethnic populations may also be involved in the incongruent results. This study evaluated most important HPA polymorphisms (HPA-1, HPA-2, HPA-3) as well as those with little considerations in previous studies (HPA-4, HPA-5, HPA-6, HPA-9, and HPA-15) in association with CAD susceptibility in an Iranian population.

## Study subjects and methods

### Patients and controls

The current retrospective case–control study, comprising 200 unrelated Iranian patients with CAD, consisted of 124 males and 76 females (mean age of 62.21 ± 9.48). The subjects were selected consecutively from individuals who referred to the Rajaei Heart Hospital, Tehran, Iran between January 2018 until August 2019. As the control group, 100 healthy and unrelated Iranian individuals, consisting of 55 males and 45 females (mean age of 57.74 ± 10.5), who attended to Masoud Medical Laboratory, Tehran, Iran for a routine checkup, were included in the study. The healthy control subjects did not have autoimmune and inflammatory diseases, cancers, metabolite disorders, and immunodeficiencies, neither in themselves nor their immediate family members.

The diagnosis of CAD was accomplished in accordance with the visual evaluation of the coronary angiogram by cardiologist. A threshold of luminal narrowing greater than 50% in diameter seen in minimum one of the main coronary arteries or related main branches was regarded as validation for diagnosis of CAD. The clinical history of patients, such as risk factors related to the cardiovascular development, was obtained from CAD patients. The scores for blood pressure, glucose, and lipid profile were obtained from the medical records at the time of diagnosis, when the subjects were detected for CAD but were not under medication. Furthermore, there was a number of subjects that were newly diagnosed, which were also not under drug regimen for controlling blood pressure, glucose, and lipid profile. The patients were assessed for the presence of diabetes mellitus based on a raised fasting blood glucose (FBS) > 130 mg/dl. Individuals with a body mass index (BMI), which was determined through body weight divided by height squared (kg/m2), of higher than 30 was considered as obese patients. To determine the hypertension in the study participants, the seated blood pressure (BP) was measured (> 140/90 mmHg on two different occasions). Hypercholesterolemia was defined as a total cholesterol level above 200 mg/dl. Baseline features of the patients and healthy control individuals are described in detail in Table 1.

**Table 1:** Characteristics of study participants

Variables	CAD patients (n = 200)	Controls (n = 100)	*P* value
Age (years) (mean ± SD)	62.21 ± 9.48	57.74 ± 10.5	0.272^a^
Gender (M/F) (%)	124 (62)/76 (38)	55 (55)/45 (45)	0.184^b^
Smokers (%)*	76 (38)	14 (14)	0.070^b^
Systolic BP (mmHg) (mean ± SD)	142.4 ± 16.91	119.3 ± 9.58	0.000^a^
Diastolic BP (mmHg) (mean ± SD)	88.7 ± 14.7	79.1 ± 11.1	0.090^a^
BMI (kg/cm^2^) (mean ± SD)	48.02 ± 4.12	27.06 ± 3.60	0.041^a^
FBS (mg/dl) (mean ± SD)	119.90 ± 43.44	95.14 ± 8.85	0.000^a^
Total Cholesterol (mg/dl) (mean ± SD)	295.39 ± 40.80	174.65 ± 27.16	< 0.001^a^
TG (mg/dl) (mean ± SD)	176.66 ± 90.84	122.91 ± 33.81	< 0.001^a^
HDL (mg/dl) (mean ± SD)	38.83 ± 9.90	57.15 ± 12.32	0.000^a^
LDL (mg/dl) (mean ± SD)	201.43 ± 24.91	88.88 ± 27.78	< 0.001^a^

*CAD* chronary artery disease, *BP* blood pressure, *BMI* body mass index, *FBS* fasting blood sugar, *TG* triglyceride, *HDL* high-density lipoprotein, *LDL* low-density lipoprotein

^a^Mann-Whitney *U* test

^b^Pearson’s chi-square test

*Both currently active and subjects with history of smoking (at least 2 years) were included

The local ethical committee of Iran University of Medical Sciences, Tehran, Iran approved the protocol of the study. Before sampling, all study participates signed the informed consent forms. Upon an overnight fasting, peripheral blood samples were obtained from all patients and control subjects in EDTA-treated vacuum tubes.

### Genotyping of HPA polymorphisms by PCR-SSP

**Fig. 1:**
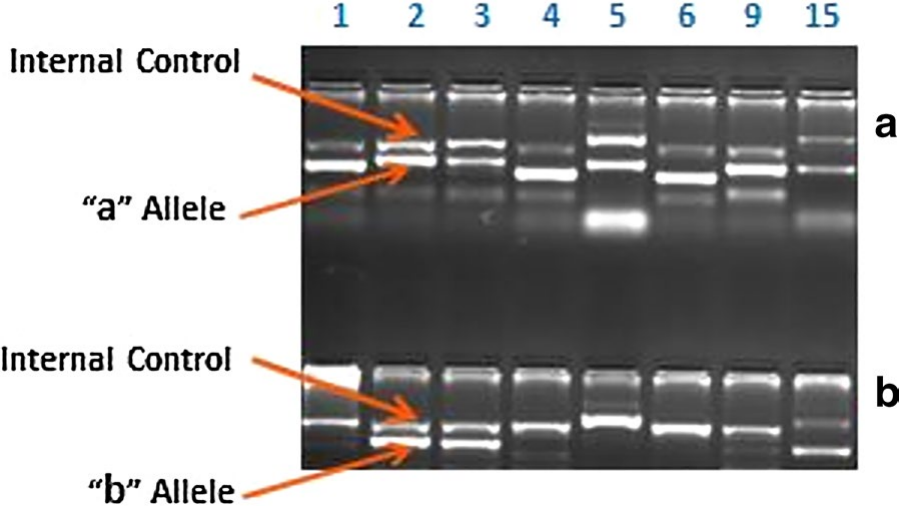
Gel electrophoresis of PCR-SSP products for HPA genotyping. Representative typing result revealing identification of homozygous a/a (HPA 1, 4, 5, 6 and 9) heterozygous a/b (HPA 2, 3 and 15). The upper arrow indicates a 429 bp of human growth hormone gene as internal amplification control for HPA 1,2,3,4,6,9 and a 607 bp of DRα gene as internal control for HPA 5 and 15, while the lower arrow indicates specific HPA allele. Full-length gel images are presented in Additional file 1: Fig. 1

The DNA content was extracted from blood samples by the salting-out approach [14]. After dissolving the DNAs with RNase-free water, samples were stored at 4 °C for pending assays. In order to genotype eight HPA polymorphisms [including HPA-1 T196C (rs5918), HPA-2 C524T (rs6065), HPA-3 T2622G (rs5911), HPA-4 G526A (rs5917), HPA-5 G1648A (rs1062535), HPA-6 A1564G (rs13306487), HPA-9 A2603G (rs137852907), and HPA15 A2108G (rs10455097)], the polymerase chain reaction with sequence-specific primers (PCR-SSP) approach was employed, as already described [15]. The detection of two different alleles through PCR-SSP technique depends on the Taq polymerase inability to repair a single base mismatch located in the 3′-end of a primer. The amplification of the target region is happened if a complementary matching of the 3′-end of primer occurred to the sequence at the allelic polymorphism region. Otherwise, mismatching of the primer and sequence of the allelic variation leads to no amplification. Wild-type and variant HPA alleles for each locus were assigned as “a” and “b”, respectively. The thermocycling program of the PCR were: 1 cycle of 95 °C for 2 min, then followed by 10 cycles of 95 °C for 10 s and 65 °C for 1 min, then 20 cycles of 95 °C for 10 s, 61 °C for 50 s and 72 °C for 30 s, finally holding at 4 °C. The thermocycling conditions of the PCR were in accordance of the previous recommendation, with slight alterations on the annealing temperature of the primers (Table 2). In order to detect each allele of the HPA polymorphisms, two sets of primers, one for allele-specific and another for the common allele, were employed (Table 2) [16]. After performing electrophoresis of the amplified samples on the agarose gel (2% w/v) stained with DNA safe stain, discrimination of the alleles was conducted through direct observation of the products. Figure 1 illustrates the electrophoretic bands related to each allele of the determined HPA polymorphisms.

**Table 2:** Primer sequences, mixes, and their specificities in PCR-SSP assay

Primers	Sequences	Annealing temperature (°C)	Primer mixes and concentrations	Amplicon size (bp)
HPA-1a HPA-1b common	5ʹ-ACTTACAGGCCCTGCCTCT-3ʹ 5ʹ-ACTTACAGGCCCTGCCTCC-3ʹ 5ʹ-AGCCGGAGTGCAATCCTCTG-3ʹ	62 64 66	HPA-1a + common, 0.5 µM HPA-1b + common, 0.5 µM	196
HPA-2a HPA-2b common	5ʹ-CCCCCAGGGCTCCTGAC-3ʹ 5ʹ-GCCCCCAGGGCTCCTGAT-3ʹ 5ʹ-GCCAGCGACGAAAATAGAGG-3ʹ	64 62 62	HPA-2a + common, 0.5 µM HPA-2b + common, 0.5 µM	241
HPA-3a HPA-3b common	5ʹ-GGGGGAGGGGCTGGGGA-3ʹ 5ʹ-GGGGGAGGGGCTGGGGC-3ʹ 5ʹ-GACCTGCTCTACATCCTGGA-3ʹ	64 66 60	HPA-3a + common, 0.5 µM HPA-3b + common, 0.5 µM	230
HPA-4a HPA-4b common	5ʹ-GCTGGCCACCCAGATGCG-3ʹ 5ʹ-AGCTGGCCACCCAGATGCA-3ʹ 5ʹ-GCTGTCCTGGCGTCTGGAG-3ʹ	62 60 62	HPA-4a + common, 0.5 µM HPA-4b + common, 0.5 µM	158
HPA-5a HPA-5b common	5ʹ-AGTCTACCTGTTTACTATCAAAG -3ʹ 5ʹ-AGTCTACCTGTTTACTATCAAAA -3ʹ 5ʹ-CTCTCATGGAAAATGGCAGTA-3ʹ	62 60 62	HPA-5a + common, 2 µM HPA-5b + common, 2 µM	249
HPA-6a HPA-6b common	5ʹ-GACGAGTGCAGCCCCCG-3ʹ 5ʹ-GGACGAGTGCAGCCCCCA-3ʹ 5ʹ-TAGCGGACACAGGAGAAGTC-3ʹ	60 62 62	HPA-6a + common, 0.5 µM HPA-6b + common, 0.5 µM	163
HPA-9a HPA-9b common	5ʹ-GGGCAGCCCCCAGTCCAC-3ʹ 5ʹ-GGGCAGCCCCCAGTCCAT-3ʹ 5ʹ-GACCTGCTCTACATCCTGGA-3ʹ	64 62 62	HPA-9a + common, 0.5 µM HPA-9b + common, 0.5 µM	212
HPA-15a HPA-15b common	5ʹ-TTCAAATTCTTGGTAAATCCTGT -3ʹ 5ʹ-TTCAAATTCTTGGTAAATCCTGG -3ʹ 5ʹ-ATGACCTTATGATGACCTATTC-3ʹ	60 62 60	HPA-15a + common, 2 µM HPA-15b + common, 2 µM	225
HGH-F HGH-R	5ʹ-GCCTTCCCAACCATTCCCTTA-3ʹ 5ʹ-TCACGGATTTCTGTTGTGTTTC-3ʹ	64 62	HGH (F + R), 0.2 µM	429
DRα-F DRα-R	5ʹ-GAGGTAACTGTGCTCACGAACAGC-3ʹ 5ʹ-CACGTTCTCTGTAGTCTCTGGG-3ʹ	74 68	DRα (F + R), 0.2 µM for HPA-5 DRα (F + R), 0.1 µM for HPA-15	607

### Statistical analysis

The gene-counting approach was used to determine the frequency of the alleles for each polymorphism of HPAs. The Fisher’s exact or Pearson’s chi-squared tests was implemented in order to determine the deviation of genotype frequencies in the control group from Hardy–Weinberg equilibrium (HWE). Univariate and then multivariate logistic linear regression analyses were employed to calculate the odds ratios (ORs) and corresponding 95% confidence intervals (CIs). The differences in the frequencies of the allele and genotype of HPA polymorphisms were determined using Fisher’s exact or Pearson’s chi-squared tests. The expectation maximization approach was carried out in order to estimate the HPA haplotypes using the online SHEsis software [17]. The correction of *P* values in more than 2 comparisons was conducted by the Bonferroni method. The relative risks were adjusted for the confounding variables, such as diabetes mellitus, hypertension, BMI, total cholesterol, and triglycerides. The difference between numerical variables was determined through Mann–Whitney *U* test to compare the means between CAD patients and healthy controls. A *P* value < 0.05 was regarded as the statistical level of significance. The data analysis was conducted using SPSS v.22 software. Presentation of data was done through mean ± standard deviation (SD) and percentage fraction of the total value for continuous and nominal variables, respectively.

## Results

### Frequency of the allele and genotypes of the HPA polymorphisms

Table 3 shows the frequency of the alleles and genotypes of each HPA polymorphism in both patient and control groups. No deviation from HWE was detected for the genotype distribution of the HPA polymorphisms in the control group.

**Table 3:** Allele and genotype distribution of HPA polymorphisms in CAD patients and healthy controls

dbSNP	Alleles/genotypes	CAD (n = 200) N (%)	Control (n = 100) N (%)	χ^2^	*P* value	OR (95% CI)
HPA1	T (a)	363 (90.7)	178 (89)	0.460	0.497	1.212 (0.69–2.11)
	C (b)	37 (9.3)	22 (11)	–	–	0.824 (0.47–1.43)
	TT (aa)	163 (81.5)	79 (78)	0.516	0.472	1.242 (0.68–2.24)
	CT (ab)	37 (18.5)	22 (22)	–	–	0.804 (0.44–1.45)
	CC (bb)	0 (0)	0 (0)	–	–	–
HWE			*P* = 0.216			
HPA2	C (a)	350 (87.5)	178 (89)	0.284	0.594	0.865 (0.50–1.47)
	T (b)	50 (12.5)	22 (11)	–	–	1.155 (0.67–1.96)
	CC (aa)	153(76.5)	79 (79)	0.303	0.859	0.865 (0.48–1.54)
	CT (ab)	44 (22)	20 (20)	–	–	1.128 (0.62–2.04)
	TT (bb)	3 (1.5)	1 (1)	–	–	1.507 (0.15–14.68)
HWE			*P* = 0.830			
HPA3	T (a)	235 (58.8)	118 (59)	0.003	0.953	0.989 (0.70–1.39)
	G (b)	165 (41.2)	82 (41)	–	–	1.010 (0.71–1.42)
	TT (aa)	74 (37)	38 (38)	0.061	0.969	0.958 (0.58–1.57)
	GT (ab)	87 (43.5)	42 (42)	–	–	1.063 (0.65–1.72)
	GG (bb)	39 (19.5)	20 (20)	–	–	0.968 (0.53–1.76)
HWE			*P* = 0.187			
HPA4	G (a)	400 (100)	200 (100)	–	–	–
	A (b)	0 (0)	0 (0)	–	–	–
	GG (aa)	200 (100)	100	–	–	–
	GA (ab)	0 (0)	0	–	–	–
	AA (bb)	0 (0)	0	–	–	–
HWE			*P* = *0*			
HPA5	G (a)	363 (90.7)	183 (91.5)	0.091	0.762	0.911 (0.49–1.66)
	A (b)	37 (9.2)	17 (8.5)	–	–	1.097 (0.60–2.00)
	GG (aa)	167 (83.5)	85 (85)	0.125	0.939	0.893 (0.45–1.73)
	AG (ab)	29 (14.5)	13 (13)	–	–	1.135 (0.56–2.29)
	AA (bb)	4 (2)	2 (2)	–	–	0.951 (0.17–5.28)
HWE			*P* = 0.1			
HPA6	G (a)	400 (100)	200 (100)	–	–	–
	A (b)	0 (0)	0 (0)	–	–	–
	GG (aa)	200 (100)	100	–	–	–
	GA (ab)	0 (0)	0	–	–	–
	AA (bb)	0 (0)	0	–	–	–
HWE			*P* = *0*			
HPA9	G (a)	400 (100)	200 (100)	–	–	–
	A (b)	0 (0)	0 (0)	–	–	–
	GG (aa)	200 (100)	100	–	–	–
	GA (ab)	0 (0)	0	–	–	–
	AA (bb)	0 (0)	0	–	–	–
HWE			*P* = *0*			
HPA15	C (a)	188 (47)	88 (44)	0.483	0.487	1.128 (0.80–1.58)
	A (b)	212 (53)	112 (56)	–	–	0.886 (0.62–1.24)
	CC (aa)	43 (21.5)	21 (21)	1.033	0.596	1.030 (0.57–1.85)
	AC (ab)	102 (51)	46 (46)	–	–	1.221 (0.75–1.97)
	AA (bb)	55 (27.5)	33 (33)	–	–	0.770 (0.45–1.29)
HWE			*P* = 0.505			

*SNP* single nucleotide polymorphism, *CAD* coronary arthery disease, *HWE* Hardy–Weinberg equilibrium

The distribution of the HPA alleles and genotypes did not show significant differences between CAD patient and healthy control groups. It was observed that the HPA-15b allele frequency was the highest in patients and healthy control subjects. The homozygous HPA-15b/15b and HPA-3b/3b genotypes were detected in high prevalence in patient and healthy control groups. The frequencies of the alleles and genotypes of HPA-4b, HPA-6b, and HPA-9b polymorphisms were not seen to have any differences between patients and controls, and therefore were omitted from further analysis (Table 3).

### Haplotype frequencies

The analysis of haplotype was based on three variants, including HPA1, HPA2, and HPA3. Among the eight identified HPA haplotypes, none of them indicated statistically significant differences between CAD patients and controls. In addition, upon performing the Bonferroni correction, it was observed that the differences were not significant for HPA haplotypes between patients and control subjects (Table 4).

**Table 4:** HPA haplotypes of HPA 1–3 in patients and controls

Haplotypes^a^			Frequencies		Association test			
HPA1	HPA2	HPA3	Hap.freq CAD (freq)	Hap.freq Controls (freq)	χ^2^	*P* value^*b*^	*Pc*^*c*^	OR (95% CI)*
a	a	a	193 (0.482)	87 (0.437)	0.947	0.330597	0.910024	1.186 (0.84–1.67)
a	a	b	125 (0.313)	73 (0.363)	1.672	0.196024	0.729941	0.789 (0.55–1.13)
a	b	a	23 (0.058)	12 (0.059)	0.007	0.931565	1	0.969 (0.47–1.99)
a	b	b	22 (0.054)	6 (0.031)	1.639	0.200514	0.738865	1.804 (0.72–4.50)
b	a	a	17 (0.042)	15 (0.073)	2.743	0.097746	0.460523	0.547 (0.26–1.12)
b	a	b	15 (0.037)	3 (0.016)	2.023	0.155039	0.636068	2.357 (0.69–7.95)
b	b	a	2 (0.006)	4 (0.020)	–	–		–
b	b	b	3 (0.008)	0 (0.000)	–	–		–

*CAD* coronary artery disease

*95% confidence interval for difference between Hap.freq case–control; If a CI does not contain 0, frequencies of haplotypes in case and control groups are significantly different at α = 0.05

^a^HPA haplotype (HPA1/HPA2/HPA3) frequency determined by the maximum likelihood method

^b^Fisher’s exact test

^c^Pc = corrected P, according to the Bonferroni method [Pc = 1 − (1 – P)^n^], where n = number of comparisons

### Regression analysis

The frequency of all potential haplotypes did not show statistically significant differences between CAD patients and healthy controls, according to the univariate regression analysis (Table 5). However, when the HPA-1a/2a/3a haplotype was considered as reference (OR = 1.00), there was a statistically significant association of HPA-1b/2a/3b (*P* = 0.015, OR = 2.792, 95% CI 1.45–8.59) with CAD risk in the multivariate regression analysis upon adjusting for the confounding factors (Table 5).

**Table 5:** Univariate and multivariate regression for haplotypes of HPA1-3 polymorphisms

	Univariate regression			Multivariate regression		
	Z-score	*P* value	OR (95% CI)	Z-score	*P* value	OR (95% CI)^a^
1a/2a/3a	–	–	1.00	–	–	1.00
1a/2a/3b	− 1.321	0.186	0.772 (0.53–1.13)	− 0.750	0.454	0.838 (0.53–1.33)
1a/2b/3a	− 0.385	0.700	0.864 (0.41–1.81)	− 0.769	0.442	0.698 (0.28–1.75)
1a/2b/3b	1.052	0.293	1.653 (0.65–4.22)	0.118	0.905	1.075 (0.33–3.54)
1b/2a/3a	− 1.782	0.075	0.511 (0.24–1.07)	− 0.943	0.345	0.645 (0.26–1.60)
1b/2a/3b	1.259	0.208	2.254 (0.64–4.99)	**2.436**	**0.015**	**2.792 (1.46–8.60)**
BMI (kg/cm^2^)	–	–	–	0.251	0.094	1.058 (0.98–1.08)
Sys.BP (mmHg)	–	–	–	2.333	0.013	1.021 (1.00–1.04)
FBS (mg/dl)	–	–	–	3.571	0.000	1.040 (1.03–1.05)
Total.Chol(mg/dl)	–	–	–	3.306	0.000	2.008 (1.00–2.02)
LDL (mg/dl)	–	–	–	− 4.633	0.001	1.967 (0.96–1.98)
TG (mg/dl)	–	–	–	2.817	0.000	1.017 (1.01–1.02)

*OR* odds ratio, *CI* confidence interval, *BP* blood pressure, *BMI* body mass index, *FBS* fasting blood sugar, *TG* trigliserid, *LDL* low-density lipoprotein

^a^Adjusted for age, BMI, systolic and diastolic BP, FBS, total cholesterol, TG, and LDL concentrations

## Discussion

Here we evaluated the association of eight HPA polymorphisms with CAD in an Iranian population. Our findings indicated that the frequencies of all HPA alleles and genotypes were not significantly different between CAD patients and healthy controls. Moreover, there was no significant association of HPA haplotypes as independent risk factors with CAD development in the Iranian population. Nonetheless, after controlling for traditional CAD risk factors, our data supported the involvement of the HPA haplotype (1b/2a/3b) with CAD risk.

A study in a Japanese population reported no association of HPA1-6 genotypes with MI, which was in line with our results [18]. Our study is also consistent with a recent Tunisian study that reported the association of haplotypes containing HPA-1b allele, such as 1b/2a/3a and 1b/2a/3b with CAD risk. This association was still significant when it was adjusted for the traditional risk factors involved in the CAD development [19]. Our findings are in line with the results of prior research performed by Floyd et al. in the UK. Their results did not find any association between HPA-1 polymorphism and CAD in patients over 45 years old. Nonetheless, the presence of the HPA-1b allele was considered as a risk factor for development of cardiovascular diseases in younger patients (≤ 45 years). It appeared that the relative effect of this polymorphism is decreased considerably with increasing in age and the presence of risk factors, such as blood pressure, diabetes, and cholesterol [20]. Nevertheless, our results are in apparent disagreement with previous reports indicating a positive association of HPA-1b and HPA-3b alleles with the risk of CAD in Tunisian patients [19], platelet hyper-reactivity in ACS [21, 22], and increased thrombotic complications [8, 23, 24].

Our study confirms the study of Kvasnicka et al*.* on HPA-1 and HPA-5 polymorphisms in a large case–control study involving 2369 cardiovascular patients with a history of the Vein thrombo embolism (VTE). Based on their statistical analysis, the allele frequency of the HPA-1 and HPA-5 polymorphisms did not associate with susceptibility to CAD [25]. This paper also proposes robust evidences along with Wei et al*.* study on HPA-1 and HPA-2 polymorphisms in Coronary heart disease (CHD) patients with more than 50% diameter stenosis. They showed that there was no relationship between the alleles and genotypes of HPA-1 and HPA-2 polymorphisms and the incidence of CAD [26]. In contrast, Zhang et al*.* proposed the HPA-2b allele as a major risk factor for CHD disease in Chinese population. They reported that the frequency of HPA-2a/2b and HPA-2b/2b genotypes had a significant relationship with the incidence of the disease. Nonetheless, a meta-analysis of 25 case–control studies demonstrated a significant association between HPA-2b allele and HPA-2a/2b and HPA-2b/2b genotypes with incidence of CAD [27, 28].

Our findings shed additional light on the conception that, whereas individual genetic variations possess a confining impact on CAD proneness, the haplotype analysis permits valid recognition of cases at high- and low-risk in the development of CAD [29, 30]. HPA-1a/1b, -2a/2b, -3a/3b are located on the GPIIIa, GPIbα, and GPIIb, respectively, which are the most frequent GPs and are important in the immunologic reactions. Genetic polymorphisms of these antigens may increase the adhesion tendency of the platelets to the harmed endothelium [11]. Therefore, specific HPA haplotypes may promote the activation, aggregation, and adhesion of platelets to the vessel endothelium during pathological conditions and the presence of risk factors like high blood pressure. These events may eventuate in the narrowing of the vessel intima and development of atherosclerotic plaques through recruitment of further inflammatory leukocytes to the site of injured endothelium [9, 10, 31]. Our analysis indicated increased risk of CAD susceptibility in cases harboring the HPA1b/HPA2a/HPA3b haplotype.

Based on our data, no associations of homozygous HPA-3b/3b genotype and HPA-3b allele were identified with predisposition to CAD in the Iranian population. These reports are in agreement with a study in German population, which indicated that there were no associations of both HPA-1 and HPA-3 variants, in the allelic, genotypic or haplotypic analysis, with risk of MI or CAD susceptibility [32]. However, it was observed that HPA-3b/b had a protective role in the young male (< 56 years of age) from Korean CAD cases [33]. By contrast, this is in apparent disagreement with reports of Lekakis et al., which indicated that HPA-3b allele and HPA-3b/3b genotype did not associate with the intensity of coronary thrombosis in patients from Greece [34]. Although these incongruities remain to be speculated, they might stem from varieties in the diagnostic criteria of the patients or differences in the number of patients or control subjects included [18, 35], as well as the differences in the ethnicity of the study participants [18, 32, 33, 36]. For instance, HPA-1b prevalence has been reported to be higher in Iranian healthy individuals (11%) than African (8%) [37], and Southeast Asians (1%) [38]. On the contrary, prevalence was lower than for Northern Europe (14–19%) [39, 40], American populations (19.1%) [24], and Tunisians (45.4%), which is the utmost prevalence identified to date for all studied population ever [19].

Our data did not support any association of HPA polymorphisms with CAD in Iranian population. However, there was an association between the HPA haplotypes and CAD susceptibility after adjusting for traditional risk factors of CAD using the regression analysis models to determine the contribution of haplotypes for CAD development. It should be noted that the patients usually undergo a drug regiment to control blood pressure, glucose, and lipid after diagnosis of CAD. We used the recorded data of the patients before initiation of medication, not follow-up period. This issue could be contributing in obtaining solid and straightforward results in the analysis that could, otherwise, confer biased outcomes.

Among the limitations and caveats of the current study were the relatively small number of subjects, the retrospective case–control design of the study, and the HPA polymorphisms analyzed. As a consequence, the interpretation of these data should be conducted cautiously considering the platelet function in CAD, as the platelet functions in healthy individuals probably differ from that of CAD patients, in which other items may also impress the physiology of platelets. We suggest a prospective study with a greater sample size for higher confidence level in approaching our results. We also suggest to investigate major adverse cardiac events within the formation of plaque in vessels and to explore their association with platelet antigens polymorphism.

## Conclusion

Considering all the results, this was the first replication study of HPA polymorphism association with CAD risk in an Iranian population. In spite of non-significant association of alleles, genotypes, and haplotypes of HPA polymorphisms with CAD risk, HPA1b/2a/3b haplotype was detected to be dependent disease risk after adjustment for confounding factors. Being armed with the comprehensive knowledge of the functional implications of the genetic polymorphism in HPAs might be useful to stratify the patients based on the inherited risk factors for thrombosis, which in turn, might be beneficial in devising and developing novel treatment options to monitor and manage the arterial thrombosis in CAD patients. To comment with certainty on the implications of the HPA polymorphisms in the pathogenesis of CAD, further independent studies in different populations exerting larger sample sizes will be contributing.

## Supplementary Information

**Additional file 1: Fig. 1**. The gele electrophorosis image of the PCR products for different samples.

## Data Availability

Not applicable.

## Abbreviations

CAD: Coronary artery disease
HPA: Human platelet alloantigens
PCR-SSP: Polymerase chain reaction-sequence-specific primers
GP: Glycoproteins
BP: Blood pressure
SD: Standard deviation
HWE: Hardy–Weinberg equilibrium
OR: Odds ratios
CI: Confidence intervals
MI: Myocardial infarction
VTE: Vein thrombo embolism
CHD: Coronary heart disease
ACS: Acute coronary syndrome

## References

[1] World Health Organization (2011). Global status report on noncommunicable diseases 2010.

[2] World Health Organization (2014). Global status report on noncommunicable diseases 2014.

[3] Libby P, Theroux P (2005). Pathophysiology of coronary artery disease. Circulation.

[4] Nordlie MA, Wold LE, Kloner RA (2005). Genetic contributors toward increased risk for ischemic heart disease. J Mol Cell Cardiol.

[5] Araujo F, Santos A, Araujo V, Henriques I, Monteiro F, Meireles E, Moreira I, David D, Maciel MJ, Cunha-Ribeiro LM (1999). Genetic risk factors in acute coronary disease. Haemostasis.

[6] Roberts R (2014). Genetics of coronary artery disease. Circ Res.

[7] Wen Y-H, Chen D-P (2018). Human platelet antigens in disease. Clin Chim Acta.

[8] Corral J, Gonzalez-Conejero R, Vicente V (2004). Genetic determinants of platelet function in thromboembolic diseases. J Biol Regul Homeost Agents.

[9] Davì G, Patrono C (2007). Platelet activation and atherothrombosis. N Engl J Med.

[10] Linden MD, Jackson DE (2010). Platelets: pleiotropic roles in atherogenesis and atherothrombosis. Int J Biochem Cell Biol.

[11] Curtis B, McFarland J (2014). Human platelet antigens–2013. Vox Sang.

[12] Fuster V, Badimon L, Badimon JJ, Chesebro JH (1992). The pathogenesis of coronary artery disease and the acute coronary syndromes. N Engl J Med.

[13] Newman PJ, Goldberger A (1991). Molecular genetic aspects of human platelet antigen systems. Bailliere's Clin Haematol.

[14] Miller S, Dykes D, Polesky H (1988). A simple salting out procedure for extracting DNA from human nucleated cells. Nucleic Acids Res.

[15] Gaudet M, Fara AG, Beritognolo I, Sabatti M (2009). Allele-specific PCR in SNP genotyping. Methods Mol Biol (Clifton, NJ).

[16] Hurd CM, Cavanagh G, Schuh A, Ouwehand WH, Metcalfe P (2002). Genotyping for platelet-specific antigens: techniques for the detection of single nucleotide polymorphisms. Vox Sang.

[17] Yong Y, Lin H (2005). SHEsis, a powerful software platform for analyses of linkage disequilibrium, haplotype construction, and genetic association at polymorphism loci. Cell Res.

[18] Hato T, Minamoto Y, Fukuyama T, Fujita S (1997). Polymorphisms of HPA-1 through 6 on platelet membrane glycoprotein receptors are not a genetic risk factor for myocardial infarction in the Japanese population. Am J Cardiol.

[19] Abboud N, Ghazouani L, Ben-Hadj-Khalifa S, Anabi F, Added F, Khalfallah A, Nsiri B, Almawi WY, Mahjoub T (2010). Human platelet alloantigens HPA-1, HPA-2, and HPA-3 polymorphisms associated with extent of severe coronary artery disease. J Thromb Thrombolysis.

[20] Floyd CN, Mustafa A, Ferro A (2014). The PlA1/A2 polymorphism of glycoprotein IIIa as a risk factor for myocardial infarction: a meta-analysis. PLoS ONE.

[21] Hackam DG, Anand SS (2003). Emerging risk factors for atherosclerotic vascular disease: a critical review of the evidence. JAMA.

[22] Davies MJ (1995). Acute coronary thrombosis–the role of plaque disruption and its initiation and prevention. Eur Heart Journal.

[23] Meisel C, Lopez JA, Stangl K (2004). Role of platelet glycoprotein polymorphisms in cardiovascular diseases. Naunyn-Schmiedeberg's Arch Pharmacol.

[24] Weiss EJ, Bray PF, Tayback M, Schulman SP, Kickler TS, Becker LC, Weiss JL, Gerstenblith G, Goldschmidt-Clermont PJ (1996). A polymorphism of a platelet glycoprotein receptor as an inherited risk factor for coronary thrombosis. N Engl J Med.

[25] Kvasnicka T, Bobcikova P, Malikova I, Hajkova J, Zima T (2015). The frequencies of ten platelet polymorphisms associated with atherosclerotic cardiovascular disease in patients with venous thromboembolism: a population-based case-control study. Hereditary Genet.

[26] Ni W, He J, Liu H, Liu T (2016). Association between platelet membrane glycoprotein polymorphisms and risk of coronary heart disease. Int J Clin Exp Med.

[27] Zhang J, Zhao L, Lv P, Liu G, Du W, Yang F, Du Y, Zhao L (2015). Association between polymorphisms of platelet membrane glycoprotein Ibα and risk of coronary heart disease in Han Chinese, Henan, China. Int J Clin Exp Pathol.

[28] Ni W, He J, Wang H, Liu T (2017). Association of platelet membrane glycoprotein HPA-2a/b, GP VI T13254C, and GP Ibα VNTR polymorphisms with risk of coronary artery disease: a meta-analysis. BioMed Res Int.

[29] Janssens AC, Pardo MC, Steyerberg EW, van Duijn CM (2004). Revisiting the clinical validity of multiplex genetic testing in complex diseases. Am J Hum Genet.

[30] Yang Q, Khoury MJ, Friedman J, Little J, Flanders WD (2005). How many genes underlie the occurrence of common complex diseases in the population?. Int J Epidemiol.

[31] Deckmyn H, Ulrichts H, Van de Walle G, Vanhoorelbeke K (2004). Platelet antigens and their function. Vox Sang.

[32] Bottiger C, Kastrati A, Koch W, Mehilli J, Seidl H, Schomig K, von Beckerath N, Schomig A (2000). HPA-1 and HPA-3 polymorphisms of the platelet fibrinogen receptor and coronary artery disease and myocardial infarction. Thromb Haemost.

[33] Park S, Park HY, Park C, Ko YG, Im EK, Jo I, Shin C, Lee JB, Shim WH, Cho SY (2004). Association of the gene polymorphisms of platelet glycoprotein Ia and IIb/IIIa with myocardial infarction and extent of coronary artery disease in the Korean population. Yonsei Med J.

[34] Lekakis J, Bisti S, Tsougos E, Papathanassiou A, Dagres N, Ikonomidis I, Soteriadou E, Tselepis AD, Goudevenos J, Kremastinos DT (2008). Platelet glycoprotein IIb HPA-3 polymorphism and acute coronary syndromes. Int J Cardiol.

[35] Anderson JL, King GJ, Bair TL, Elmer SP, Muhlestein JB, Habashi J, Carlquist JF (1999). Associations between a polymorphism in the gene encoding glycoprotein IIIa and myocardial infarction or coronary artery disease. J Am Coll Cardiol.

[36] Lagercrantz J, Bergman M, Lundman P, Tornvall P, Hjemdahl P, Hamsten A, Eriksson P (2003). No evidence that the PLA1/PLA2 polymorphism of platelet glycoprotein IIIa is implicated in angiographically characterized coronary atherosclerosis and premature myocardial infarction. Blood Coagul Fibrinolysis Int J Haemost Thromb.

[37] Halle L, Bigot A, Mulen-Imandy G, M'Bayo K, Jaeger G, Anani L, Martageix C, Bianchi F, Julien E, Kaplan C (2005). HPA polymorphism in sub-Saharan African populations: Beninese, Cameroonians, Congolese, and Pygmies. Tissue Antigens.

[38] Seo DH, Park SS, Kim DW, Furihata K, Ueno I, Han KS (1998). Gene frequencies of eight human platelet-specific antigens in Koreans. Transfus Med.

[39] Jones DC, Bunce M, Fuggle SV, Young NT, Marshall SE (2003). Human platelet alloantigens (HPAs): PCR-SSP genotyping of a UK population for 15 HPA alleles. Eur J Immunogenet.

[40] Kekomäki S, Kyllönen L, Salmela K, Koskimies S, Kekomäki R (2001). Platelet-specific alloantigens in cadaveric renal transplantation. A prospective study. Effect of HPA-5b mismatch in acute vascular rejection of renal allografts. Tissue Antigens.

