# Supplementary material for: Association of human platelet alloantigens encoding gene polymorphisms with the risk of Coronary artery disease in Iranian patients
Source: BMC Cardiovasc Disord. 2021 Feb 2;21:68. doi: 10.1186/s12872-021-01892-z (PMC7856748; doi:10.1186/s12872-021-01892-z)

**Supplementary Figure 1**. The gele electrophorosis image of the PCR products for different samples.


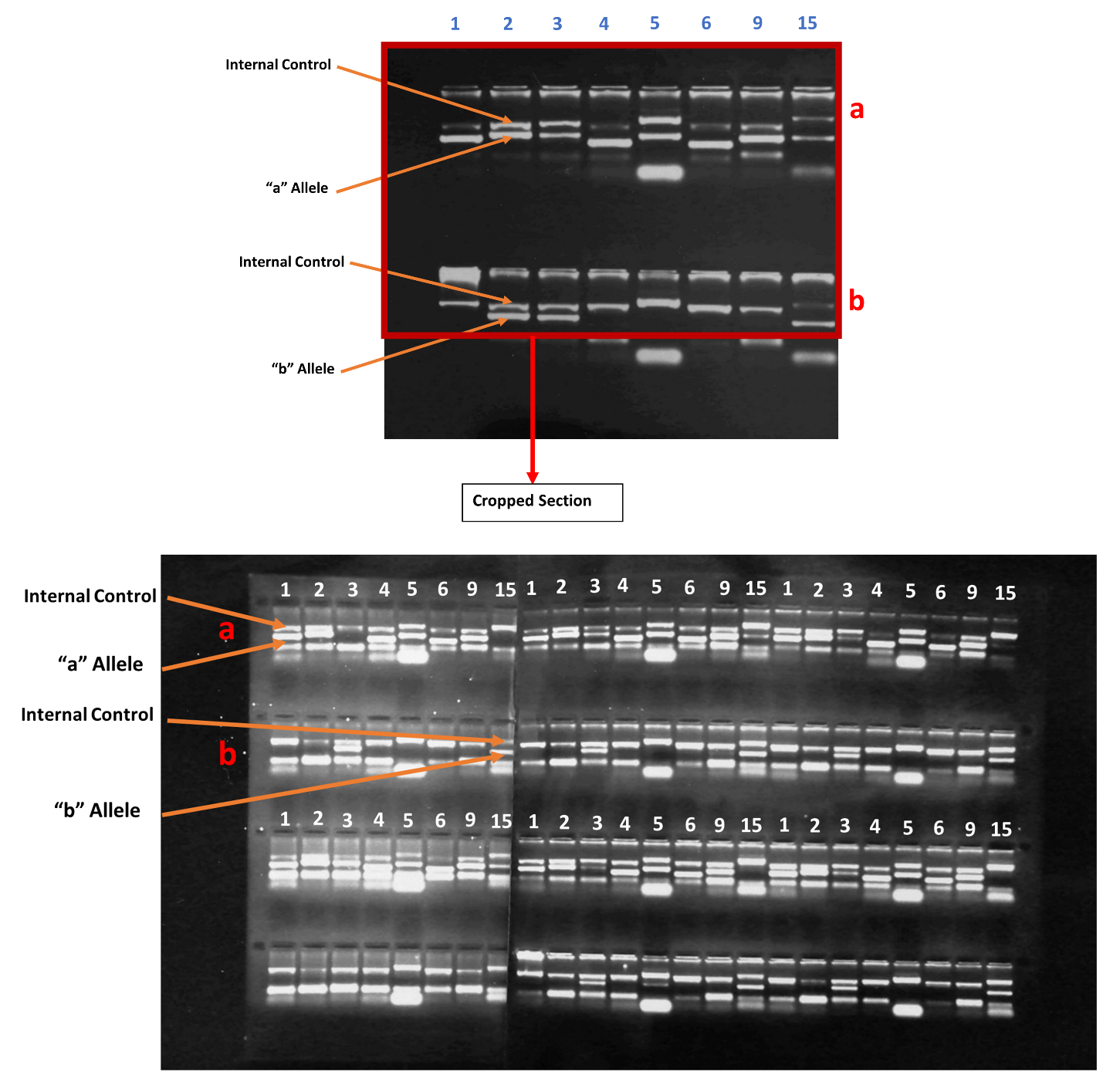

Supplement: Supplementary file 1 — Additional file 1: Fig. 1. The gele electrophorosis image of the PCR products for different samples. [file 12872_2021_1892_MOESM1_ESM.docx]
